# A DAF-3 co-Smad molecule functions in *Haemonchus contortus* development

**DOI:** 10.1186/s13071-019-3855-3

**Published:** 2019-12-27

**Authors:** Wenda Di, Lu Liu, Ting Zhang, Fangfang Li, Li He, Chunqun Wang, Awais Ali Ahmad, Mubashar Hassan, Rui Fang, Min Hu

**Affiliations:** 1 State Key Laboratory of Agricultural Microbiology, Key Laboratory of Development of Veterinary Products, Ministry of Agriculture, College of Veterinary Medicine, Huazhong Agricultural University, Wuhan, 430070 Hubei China; 2 School of Basic Medical Sciences, Hubei University of Medicine, Shiyan, 442000 Hubei China

**Keywords:** co-Smad, *daf-3*, *Haemonchus contortus*, Immunohistochemistry, RNAi, siRNA, TGF-β signalling

## Abstract

**Background:**

The Smad proteins function in TGF-β signalling transduction. In the model nematode *Caenorhabditis elegans*, the co-Smad, DAF-3 mediates R-Smads and performs a central role in DAF-7 signal transduction, regulating dauer formation and reproductive processes. Considering the divergent evolutionary patterns of the DAF-7 signalling pathway in parasitic nematodes, it is meaningful to explore the structure and function of DAF-3 in parasitic nematodes, such as *Haemonchus contortus*.

**Methods:**

A *daf-3* gene (*Hc-daf-3*) and its predicted product (*Hc*-DAF-3) were identified from *H. contortus* and characterised using integrated genomic and genetic approaches. In addition to immunohistochemistry employed to localise *Hc*-DAF-3 within adult worm sections, real-time PCR was conducted to assess the transcriptional profiles in different developmental stages of *H. contortus* and RNA interference (RNAi) was performed *in vitro* to assess the functional importance of *Hc-daf-3* in the development of *H. contortus*.

**Results:**

*Hc-*DAF-3 sequences predicted from *Hc-daf-3* displayed typical features of the co-Smad subfamily. The native *Hc*-DAF-3 was localised to the gonad and cuticle of adult parasites. In addition, *Hc-daf-3* was transcribed in all developmental stages studied, with a higher level in the third-stage larvae (L3) and adult females. Moreover, silencing *Hc-daf-3* by RNAi retarded L4 development.

**Conclusion:**

The findings of the present study demonstrated an important role of *Hc*-DAF-3 in the development of *H. contortus* larvae.

## Background

Members of the Smad protein family are critical components that transmit extracellular signals from the cell surface into the nucleus. Three distant classes of Smads have been defined including the R-Smads, the co-Smads and the I-Smads [1, 2]. In vertebrates, the bone morphogenetic protein (BMP) and TGF-β/Activin pathways share only one common co-Smad, Smad4 (DPC4), which functions in the downstream pathways. In brief, activated type I receptor phosphorylates downstream R-Smads, which results in the latter dissociating from the receptor and forming a heteromeric complex with co-Smad. Then, this heteromeric complex moves to the nucleus, where Smads can regulate transcriptional responses by targeting various DNA-binding proteins [3]. In contrast, the I-Smads function as antagonists of TGF-β signalling through preventing the phosphorylation of respective R-Smads by binding to the receptor complexes or preventing the formation of heteromeric complex with co-Smads by binding to phosphorylated R-Smads. In addition, TGF-β family members can stimulate the transcription of the I-Smad genes, offering potential negative feedback of the pathway [1, 4].

Unlike vertebrates, co-Smads are pathway-specific in DAF-7 and DBL-1 pathways of the TGF-β signalling pathway in the model nematode *Caenorhabditis elegans* [5]. In *C. elegans*, while *Ce*-SMA-4 functions in DBL-1 pathway signal transduction, another co-Smad, *Ce*-DAF-3 transduces signals in the DAF-7 pathway. The latter pathway regulates the recovery from dauer as well as bypassing dauer in reproductively favourable environments [6]. In this pathway, DAF-7 signals are transduced by *Ce*-DAF-8 and *Ce*-DAF-14 R-Smads. When both R-Smads are activated, they inhibit the functions of *Ce*-DAF-3, which promotes recovery from dauer. However, when both R-Smads are not activated, *Ce*-DAF-3 can bind to *Ce*-DAF-5 (a transcriptional factor) alone and promote the worm’s entry into dauer stage.

In *C. elegans*, the function of *Ce*-DAF-3 is negatively regulated by the upstream R-Smads, which is quite different from the typical TGF-β signaling pathways in other systems where co-Smad is positively transduced by the upstream R-Smad [7]. In addition, as there are no I-Smad in the DAF-7 signalling pathway in *C. elegans*, *Ce*-DAF-3 also functions similar to I-Smad by repressing the transcription of *Ce-daf-7* and *Ce-daf-8*, which drives a negative feedback loop of the pathway [7], though this antagonistic interaction differs from the mechanism of I-Smad’s inhibitory function in other systems.

Dauer formation is a temperature-sensitive process. However, depending on the environmental and genetic context, *Ce*-DAF-3 can function to promote or to inhibit dauer arrest. For example, *Ce-daf-3* loss-of-function mutations are Daf-d (dauer formation defective) at 25 °C [6], but Daf-c (dauer formation constitutive) at 27 °C [8], indicating that the activity of *Ce*-DAF-3 is influenced by temperature. In addition, at 25 °C, *Ce*-*daf-3* mutation suppresses dauer arrest in the Daf-c mutants *daf-1*, *4*, *7*, *8* and *14* [6] but enhances the weak dauer arrest in the Daf-c mutant (*sdf-9* mutant) [9]. Moreover, *Ce*-DAF-3 also functions in egg-laying as *Ce-daf-3* mutation can suppress the partial retention of embryos in the uterus, which is induced by the lack of DAF-7 signalling [10].

The dauer hypothesis posits that the molecular pathways that control the entry into and recovery from the dauer stage of *C. elegans* is functionally analogous to the pathways controlling the infective larval arrest and activation of parasitic nematodes [11]. Research on DAF-7 signalling pathway components in parasitic nematodes has indicated that the dauer hypothesis should be treated prudently, as it may not be suitable for DAF-7 signalling pathway exploration in parasitic nematodes [11–13]. While previous studies proposed the sequences and functions of DAF-7 signalling pathway components, a TGF-β type I receptor-like molecule was less conserved relative to its *C. elegans* homologues [14–18], suggesting the importance of studying the functions of homologues in this pathway in parasitic nematodes. Recently, we functionally characterised a TGF-β type I receptor-like molecule, *Hc-tgfbr1* in *Haemonchus contortus* (see [19]), but nothing is known about the *Ce*-DAF-3 homologue of *H*. *contortus* despite its importance in *C. elegans*.

Herein, we carried out a structural and functional study on *Hc-daf-3*, a gene encoding a co-Smad molecule of *H. contortus* (*Hc*-DAF-3) employing molecular and functional genomics techniques. Transcription levels of *Hc-daf-3* throughout the developmental stages were investigated, and the localisation of *Hc*-DAF-3 was detected in adult worms by immunohistochemistry. Furthermore, the functional importance of *Hc-daf-3* was assessed by RNA interference (RNAi) *in vitro* in *H. contortus*.

## Methods

### The maintenance of *H. contortus*

The *H. contortus* Haecon-5 strain was maintained by serial passage in 3–6 months old goats. Briefly, 3-month-old lambs that had been dewormed and maintained under parasite-free conditions were infected by oral administration of 8000 infective third-stage larvae (L3) of *H. contortus* (Hacon-5 strain). Eggs were recovered from fresh faeces of infected goats using sucrose flotation and differential sieving procedures [20]. Faeces were incubated in culture at 27 °C for 1 day, 3 days, 7 days to recover the free-living larval stages, including first-stage larvae (L1), second-stage larvae (L2) and third-stage larvae (L3). All larvae were counted and immediately frozen in liquid nitrogen for RNA isolation. Fourth-stage larvae (L4) were isolated from the abomasa (suspended in 0.5% NaCl at 40 °C for 3–5 h) of goats at day 8 post-infection. Adult worms were harvested 30 days post-infection from the abomasa of goats. The worms (L4s and adults) were rinsed, sexed, counted and frozen in liquid nitrogen.

### RNA and cDNA preparation

Total RNA was extracted from different stages/sexes of *H. contortus* using Trizol (Simgen, Hangzhou, China), and integrity and yields were examined by electrophoresis and spectrophotometry, respectively. Isolated RNA was stored at − 80 °C for subsequent reverse transcription. Complementary DNA (cDNA) was synthesised from extracted total RNA (1 µg) using PrimerScirpt^TM^ reagent kit with gDNA Eraser (Takara, Dalian, China); then, cDNA was used as template for coding sequence (CDS) amplification and real-time PCR.

### Isolation of *Hc-daf-3* CDS

Based on the transcriptomic and genomic datasets for *H. contortus* [21, 22], together with the CDS of *Ce-daf-3*, both the CDS and corresponding genomic sequence of *Hc-daf-3* were retrieved (GenBank: MK159304). The CDS of *Hc-daf-3* was amplified from cDNA with primer pair Hc-daf-3-cF/Hc-daf-3-cR (Additional file 1: Table S1) using the following protocol: 95 °C for 5 min, followed by 35 cycles of 95 °C for 30 s, 60 °C for 30 s, 72 °C for 2 min; and a final extension step at 72 °C for 10 min. The PCR product was inserted into the pTOPO Blunt cloning plasmid (Aidlab, Beijing, China) and sequenced directly with primers from both directions (*via* Tskingke Biology Technology, Wuhan, China).

### Bioinformatics analyses

Nucleotide (nt) sequences and amino acid sequences were assembled and aligned using the programs BLASTx and Clustal W [23]. In brief, the CDS sequence of *Hc-daf-3* was compared with sequences in non-redundant databases using the BLASTx from the National Center for Biotechnology Information (NCBI) (http://www.ncbi.nlm.gov/BLAST), to confirm the identity of the obtained gene sequences. The cDNA sequence of *Hc-daf-3* was conceptually translated into predicted amino acid sequences using the software DNAstar (http://www.dnastar.com). Exon and intron boundaries in *Hc-daf-3* genomic DNA sequence were retrieved from GenBank (GenBank: LS997567.1).

Additionally, the sequence of *Hc*-DAF-3 was aligned with a panel of selected reference sequences and phylogenetic trees were constructed using MEGA 6.0 [24]. In detail, the sequence of *Hc*-DAF-3 was aligned with SMAD4 amino acid sequences of *Homo sapiens* and *Mus musculus* (Additional file 1: Table S2) using BioEdit according to these two reference sequences to identify and designate functional domains, then these domains were labelled using Photoshop CS 6.0. For phylogenetic analyses, the *Hc*-DAF-3 sequence together with 19 homologous sequences selected from nine nematodes (*Ancylostoma ceylanicum*, *Ascaris suum*, *Brugia malayi*, *C. elegans*, *Caenorhabditis brenneri*, *Caenorhabditis briggsae*, *Caenorhabditis remanei*, *Loa loa* and *Toxocara canis*) and four other metazoans (*Drosophila melanogaster*, *Homo sapiens*, *Mus musculus* and *Schistosoma mansoni*) were aligned (Additional file 1: Table S2). An R-Smad, *Ce*-DAF-8, was used as the outgroup. Phylogenetic analyses of aligned sequence data were conducted using the neighbor-joining (NJ), maximum parsimony (MP) and maximum likelihood (ML) methods employing the Jones–Taylor–Thornton (JTT) model in MEGA 6.0. Confidence limits were evaluated using a bootstrap procedure with 1000 pseudoreplicates. A 50% cut-off value was implemented for the consensus tree.

### Transcriptional analysis of *Hc-daf-3* in different developmental stages

Transcriptional profiles of *Hc-daf-3* were examined by real-time PCR with the specific primers Hc-daf-3-qF/Hc-daf-3-qR (Additional file 1: Table S1) at eight developmental stages/sexes of *H. contortus* including eggs, L1s, L2s, L3s, L4 females and L4 males as well as adult females and adult males. In brief, total RNA was extracted individually from eight developmental stages/sexes of *H. contortus* using Trizol reagents and cDNA was obtained using the PrimeScript RT reagent Kit (Takara). Subsequently, real-time PCR was performed using an ABI 7100 thermal cycler to determine the gene transcriptional levels, and the reaction procedure was as following: at 95 °C for 30 s, followed by 40 cycles of 95 °C for 15 s, 60 °C for 15 s and 72 °C for 20 s. Finally, the following conditions (95 °C for 15 s, 60 °C for 1 min, 95 °C for 15 s and 60 °C for 15 s) were employed to generate the dissociation curve. Then, the mean threshold cycle values were used for the analysis. Statistical analysis was conducted using a one-way ANOVA, and *P* ≤ 0.05 was set as the criterion for significance.

### Polyclonal antibody preparation using synthetic peptides

Polyclonal antibody was produced using a synthetic peptide as antigen to immunise rabbits. Briefly, specific amino acid sequences (T1: CRRRNKSISETKR and T2: CHYLDRESGRST) of *Hc*-DAF-3 were initially selected after analysis and synthesised. The purified peptide was coupled to keyhole limpet haemocyanin (KLH), and rabbits were immunised to produce specific polyclonal antibodies against *Hc*-DAF-3. Two rabbits were injected subcutaneously at multiple sites with 400 μg of purified synthetic peptide in Freund’s complete adjuvant (four immunisations, five weeks apart). Prior to the first injection, a pre-bleed was taken from each rabbit, which was designated the ‘negative’ serum. A final bleed was taken one week after the last immunisation, and was designated the ‘positive’ serum. Sera were prepared according to a standard procedure [25]. Both positive and negative control sera (both at 1:1000 dilution) were assessed on a Western blot.

### Western blot analysis

For Western blotting, protein extracts were prepared as follows. Fresh adult worms of *H. contortus* were homogenised to 100 μl with phosphatase inhibitor and protein lysate (Roche Molecular Biochemicals, Basel, Switzerland). Solubilised protein fractions were separated by centrifugation at 10,000×*g* for 3 min at 4 °C. Lysates were stored at − 80 °C after protease inhibitor (Thermo Fisher Scientific, Waltham, USA) was added until subjected to Western blotting. Extracts were fractionated using 15% SDS-PAGE (polyacrylamide gel electrophoresis) and then electro-transferred onto a PVDF membrane. After being blocked overnight in TBST (TBS and 20% Tween-20), the blot strips were probed with the antiserum for 2 h. Then goat anti-rabbit IgG antibody (Beyotime, Shanghai, China) was incubated as a secondary antibody for another 1 h. Recognised antigens were visualised with ECL (Solarbio, Beijing, China) within 3–5 min and imaged using a chemiluminescence imaging system (Bio-Rad, Shanghai, China).

### Localization of *Hc*-DAF-3 in the adult *H. contortus* by immunohistochemistry

Approximately 50–100 *H. contortus* adults harvested from the abomasa of infected goats were washed with physiological saline for five times and fixed in 4% paraformaldehyde (Biosharp, Shanghai, China) at 4 °C for 3 days. Single female and male worms were dehydrated separately in a graded ethanol series and embedded in paraffin. Sections (4 μm) were cut using a microtome and mounted onto polysine slides. Afterwards, paraffinised (xylene-treated 2 times for 20 min) sections were rehydrated and then washed five times (5 min each) in phosphate-buffered saline (PBS). A microwave was used for antigen recovery, and 3% hydrogen peroxide was used to reduce the non-specific staining by endogenous catalase. The slides were washed with PBS (5 min) for five times and blocked with 5% w/v bovine serum albumin (BSA) for 20 min in a humidified chamber. A volume of 50 μl of the ‘positive’ or ‘negative’ serum (each at 1:100 dilution) was incubated at 4 °C overnight, respectively. Serum was removed, and slides were washed with PBS (5 min) for 3 times, followed by incubation at 37 °C for 50 min in goat anti-rabbit immunoglobulin (IgG, 1:1000) conjugated with fluorescein (Abcam, Wuhan, China) in the dark. The secondary antibody was removed, and slides were washed with PBS (5 min each) for 3 times, followed by incubation at room temperature for 5 min in 4, 6-diamidino-2-phenylindole (DAPI) solution in the dark. The sections were washed again in the same way and then examined using an epifluorescence microscope (Olympus IX-51, Tokyo, Japan). All images were visualized with Photoshop CS6.0.

### RNA interference in *H. contortus*: preparation and implementation

The CDS encoding *Hc*-DAF-3 was used to design the siRNA (small interfering RNA) sequences by the siRNA Design Tools program (https://rnaidesigner.thermofisher.com). Then, siRNA oligos were synthesised by Shanghai GenePharma Co. Ltd., while negative control siRNA oligos were also synthesised, which showed no sequence identity to any *H. contortus* nucleotide sequence. Sequences of siRNA oligos are shown in Additional file 1: Table S3 (5′ to 3′ sequence). All siRNAs were dissolved in DEPC-treated water and stored at 50 μM at − 80 °C until use. For RNAi in *H. contortus*, three siRNAs (S1 siRNA, S2 siRNA and S3 siRNA) were mixed with equimolar quantities at 1 μM for each single siRNA, and negative control was 3 μM.

Approximately 20,000 fresh L3s were collected from faeces and exsheathed in 1% hypochlorite at 38 °C for 30 min. Then, the exsheathed L3 larvae (xL3) were washed five times in sterile PBS by centrifugation at 600× *g* for 5 min at room temperature. xL3s were suspended in EBSS (Earleʼs balanced salt solution, pH 5.2, Sigma-Aldrich, St Louis, USA) containing 2.5 μg/ml of amphotericin, 100 μg/ml of streptomycin and 100 IU/ml of penicillin (Gibco, Grand Island, USA) and allocated into 96-well plates with 60 µl per well (*n* = 6000) [19]. In addition, nuclease-free water (blank control), NC siRNA (negative control, 4.8 µl) and *Hc-daf-3* siRNA (1.6 µl for each siRNA) were incubated with RNasin (0.2 µl, 40 U, Thermo Fisher Scientific) and Lipofection Reagent (5 µl, Invitrogen, Carlsbad, USA) for 20 min at 25 °C after adjusting the total volume to 20 µl. Then, the liposome-formulated water or siRNA (20 μl) was added into the 96-well plates containing xL3 as described above and incubated at 38 °C in 20% CO_2_ for 3 days. Larvae (*n* = 300) were transferred to 100 μl EBSS and incubated for a further 5 days, and the developmental rate of xL3 to L4 was examined by microscopy, while L4 development was assessed according to the morphological changes occurred in the buccal region of *H. contortus* worms [26, 27]. In addition, the remaining larvae were collected for RNA extraction and subsequent assessment of the transcriptional level by real-time PCR. The *Hc*-*18S* gene was used as a reference for calculating the relative transcriptional level. The two sets of primers (Hc-daf-3-rtF/Hc-daf-3-rtR and Hc-18S-qF/ Hc-18S-qR) for real-time PCR are shown in Additional file 1: Table S1. The PCR cycling protocol was: 95 °C for 30 s, followed by 95 °C for 15 s, 60 °C for 15 s, and 72 °C for 20 s for 40 cycles. The data were subjected to analysis using 2^−ΔΔCq^ method [28]. All experiments were repeated at least three times on different days.

## Results

### Characterisation of *Hc-daf-3* and *Hc*-DAF-3

The CDS of *Hc-daf-3* was 2097 bp in length and inferred to encode a protein of 698 amino acids with 78.62 KDa (GenBank: MK159304). The inferred amino acid sequence identities of *Hc*-DAF-3 to SMAD4 homologues from various organisms ranged between 31.4–76.5% (Additional file 1: Table S4), including *A. ceylanicum*, *T. canis*, *A. suum*, *B. malayi*, *H. sapiens*, *M. musculus*, *D. melanogaster*, *H. contortus*, *C. elegans* and *S. mansoni*. For *A. ceylanicum*, both MH1 and MH2 domains have high identities to those of *Hc*-DAF-3. However, except for *A. ceylanicum*, these two domains (MH1 and MH2 domains) of co-Smad homologues from the other organisms showed variable identities to those of *Hc*-DAF-3. Comparisons also revealed that MH1 domain has higher identities (53.6–77.1%) than the MH2 domain (35.0–45.5%) (Additional file 1: Table S4).

**Fig. 1:**
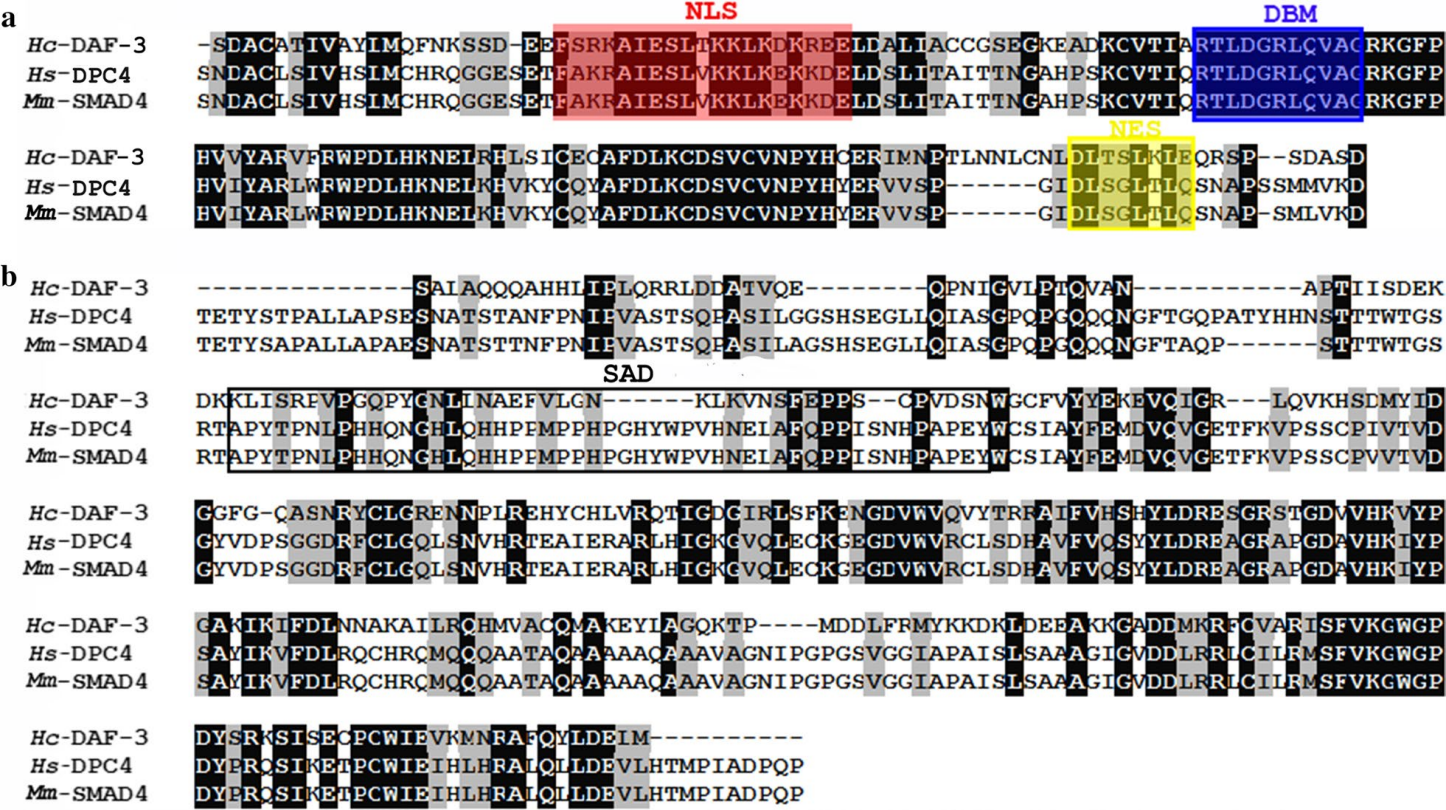
Amino acid sequence alignments for *Hc*-DAF-3 and homologues from *Homo spaiens* (*Hs*) and *Mus musculus* (*Ms*). **a** Alignments of the MH1 domain and the N-terminal sequence of the linker region. Abbreviations: NLS, nuclear localization signal; DBM, DNA-binding motif; NES, nuclear export signals. **b** Alignment of the C-terminal sequence of the linker region and the MH2 domain. Boxed sequence represent Smad4 activation domain (SAD). Marked in colour are NLS (red), DBM (blue) and NES (yellow). Consensus residues are marked in black; similar residues are marked in grey

Smad4 homologues from *H. sapiens* and *M. musculus* were aligned with *Hc*-DAF-3 to define areas with high conservation (Fig. 1). Alignments revealed that *Hc*-DAF-3 contains typical co-Smad features, including the nuclear localization signals (NLS), the DNA-binding motif (DBM) in the MH1 domain and the nuclear export signals (NES) in the linker region (Fig. 1a). NLS and NES are well conserved with all basic and leucine residues retained, but the transcriptional activation domain (SAD) shows less conservation (Fig. 1b).

### Genetic relationship of *Hc*-DAF-3 with co-Smad orthologues from 14 selected species

**Fig. 2:**
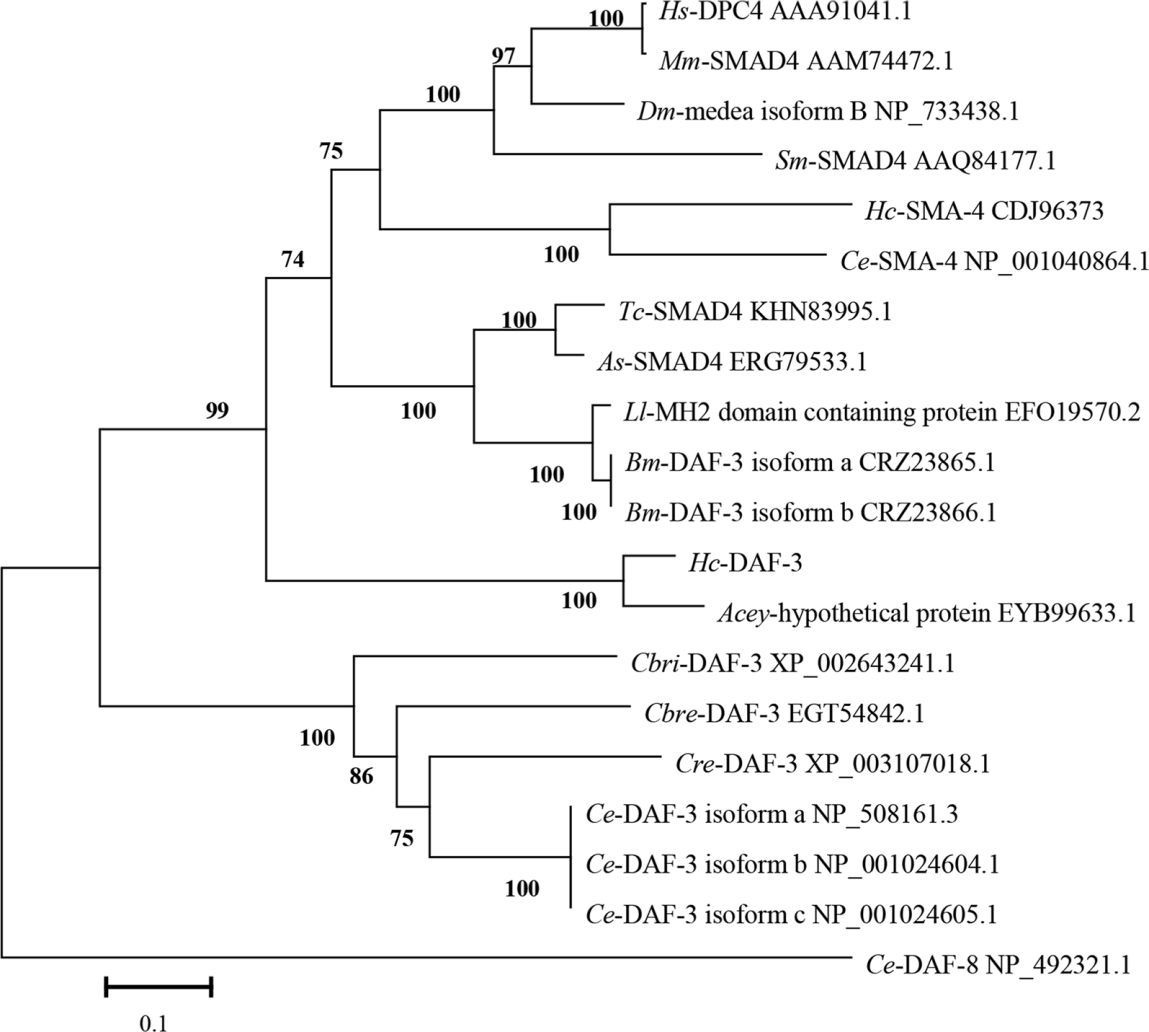
Phylogenetic relationships of *Hc*-DAF-3 and co-Smad molecules of 14 species. The neighbour-joining tree was constructed and bootstrap values are shown above or below the branches

Phylogenetic trees were constructed based on the amino acid sequences of *Hc*-DAF-3 and co-Samd homologues from 14 selected species, including nematodes (*A. ceylanicum*, *A. suum*, *B. malayi*, *C. brenneri*, *C. briggsae*, *C. elegans*, *C. remanei*, *H. contortus*, *L. loa* and *T. canis*), platyhelminthes (*S. mansoni*), arthropods (*D. melanogaster*) and chordates (*H. sapiens* and *M. musculus*). The analysis revealed that there was concordance in topology among the MP, ML and NJ trees. All co-Smads formed two large branches with high bootstrap support (99% and 100%), one contained 13 co-Smads including three from vertebrate species, one from trematode parasite and seven from parasitic nematodes, and the other contained six DAF-3s from free-living nematodes belonging to the genus *Caenorhabitis*. *Hc*-DAF-3 grouped together with *Acey*-hypothetical protein with 100% bootstrap support. Compared with *Ce*-SMA-4 and *Hc*-SMA-4, *Ce*-DAF-3 and *Hc*-DAF-3 showed a more distant relationship to *Hs*-SMAD4 (also known as *Hs*-DPC4). However, *Hc*-DAF-3 showed a closer relationship to DAF-3 homologues of parasitic nematodes, including *T. canis, A. suum*, *L. loa* and *B. malayi* (Fig. 2).

### Genomic structure of *Hc-daf-3*

**Fig. 3:**
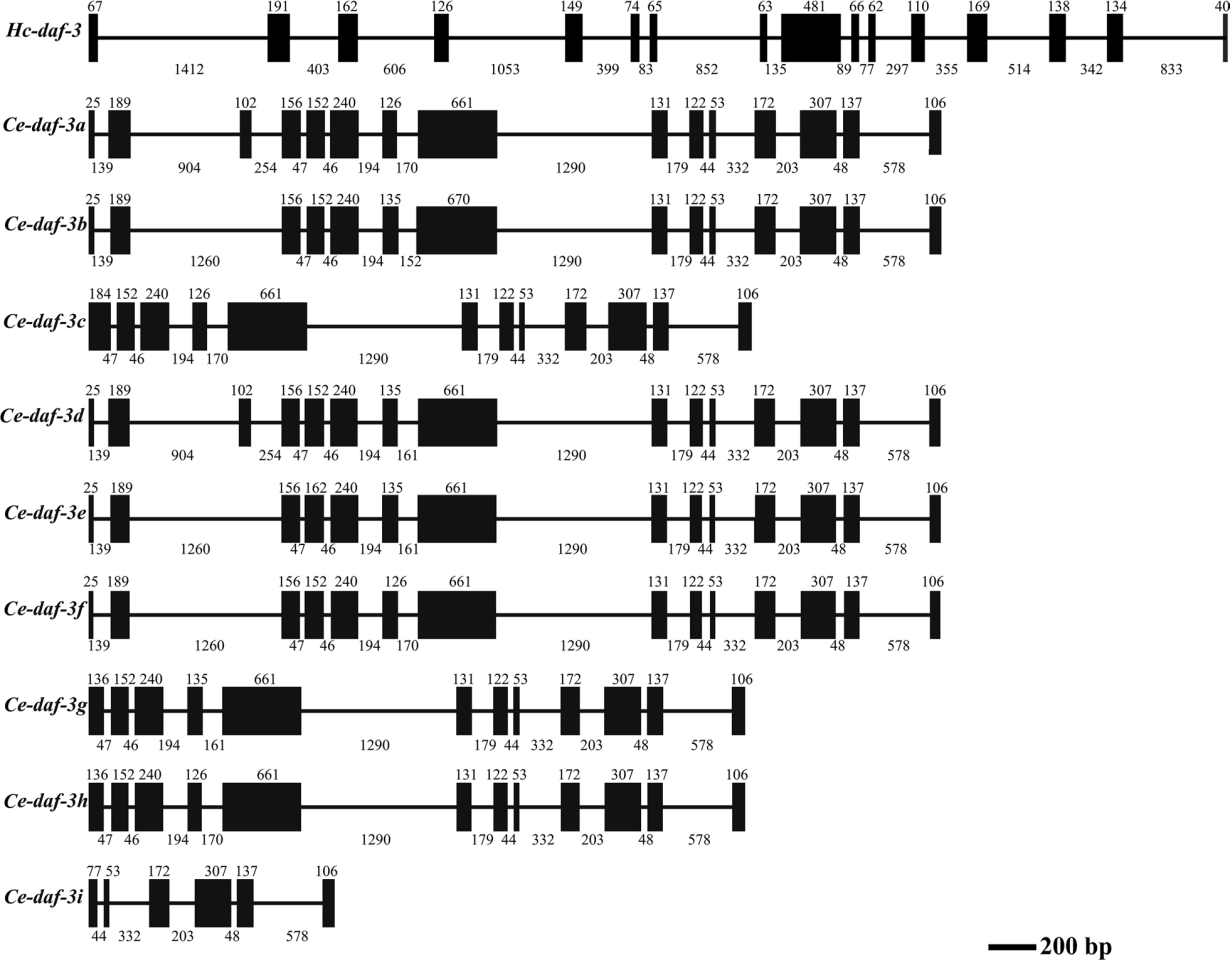
Gene structure of *Hc-daf-3* and *Ce-daf-3*. Black boxes represent exons, and the horizontal lines represent introns. The numbers indicate the corresponding lengths of exons and introns

The full length gDNA of *Hc-daf-3* was 9487 bp and contained 16 exons with the lengths ranging between 40–481 bp and 15 introns with the lengths ranging between 77–1412 bp; it had more exons and introns than *Ce-daf-3*, which had nine transcripts with 6 to 15 exons (Fig. 3). Although nine transcripts were found in *C. elegans*, only one was identified in *H. contortus*. For *Ce-daf-3*, while transcripts a/b/d/e/f had 14–15 exons, transcripts c/g/h had 12 exons. However, transcript i only has 6 exons (Fig. 3).

### Transcriptional profiles of *Hc-daf-3* in *H. contortus*

**Fig. 4:**
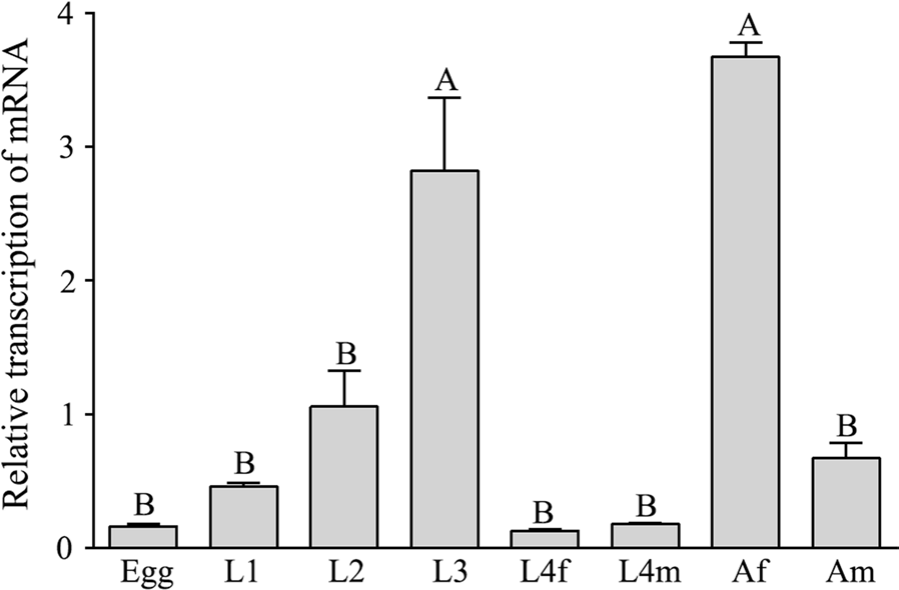
The transcription of the *Hc-daf-3* gene in eight developmental stages of *Haemonchus contortus*. The relative quantities (compared with L2, L2 = 1) are shown as the mean values (+ standard error of the mean, SEM) derived from three replicates in repeat experiments. The significant differences between stages are indicated by different letters, while the same letter for different stages indicates no difference

The transcription of *Hc-daf-3* was detected at eight developmental stages/sexes including eggs, L1s, L2s, L3s, female L4s, male L4s, female adults and male adults (Fig. 4). Compared with other stages, the transcriptional levels in L3 and adult female were significantly higher (*F*_(7, 15)_ = 29.27, *P* < 0.01), whereas there was no significant difference among the other stages.

### Expression pattern of *Hc*-DAF-3 in *H. contortus*

An anti-*Hc*-DAF-3 polyclonal antibody was produced by immunising rabbit with synthetic peptides. This antibody can specifically bind native *Hc*-DAF-3 in whole-worm extracts of *H. contortus* detected by Western blot as a single band of ~ 79 kDa, consistent with the molecular mass of native *Hc*-DAF-3 protein (Additional file 2: Figure S1).

**Fig. 5:**
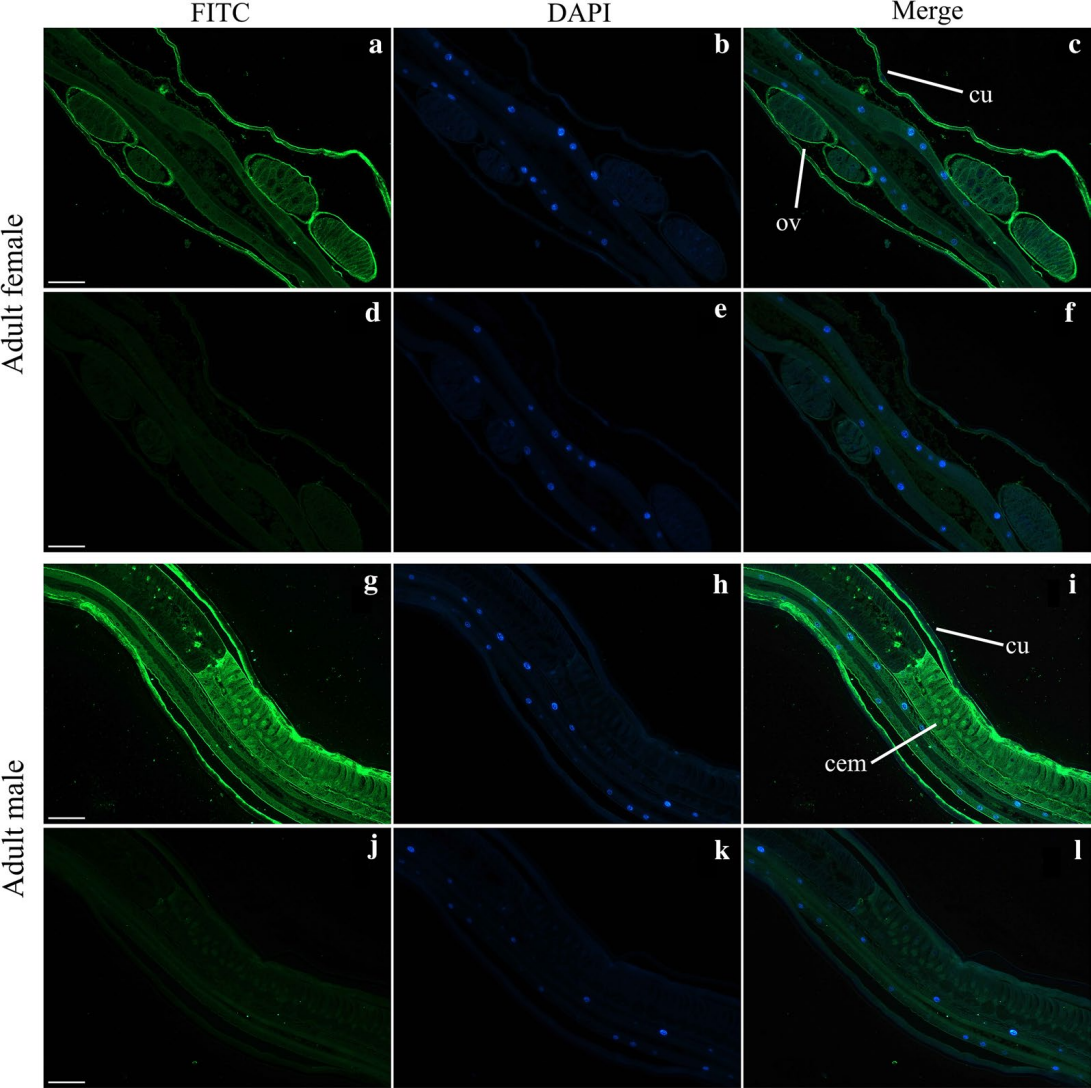
Localisation of *Hc*-DAF-3 in adult *Haemonchus contortus* by immunohistochemistry. **a**–**f** Localisaion of *Hc*-DAF-3 in *H. contortus* adult females. **g**–**l** Localisation of *Hc*-DAF-3 in *H. contortus* adult males. Abbreviations: cem, cement gland; cu, cuticle; ov, ovary. *Scale-bars*: 100 µm

The expression of *Hc*-DAF-3 in adult worms of *H. contortus* was then examined using this anti-*Hc*-DAF-3 antibody (Fig. 5). In addition to the cuticle of both female and male worms, expression was detected in the gonad of adult females, especially in the ovary wall (Fig. 5a–c). Native *Hc*-DAF-3 was also localised in the cement gland of adult males (Fig. 5g–i). The cement gland is the sexual tube connecting the vesicula seminalis with the cloaca [29].

### Assessment of the effect of *Hc-daf-3*-specific siRNA on larval development of *H. contortus*

**Fig. 6:**
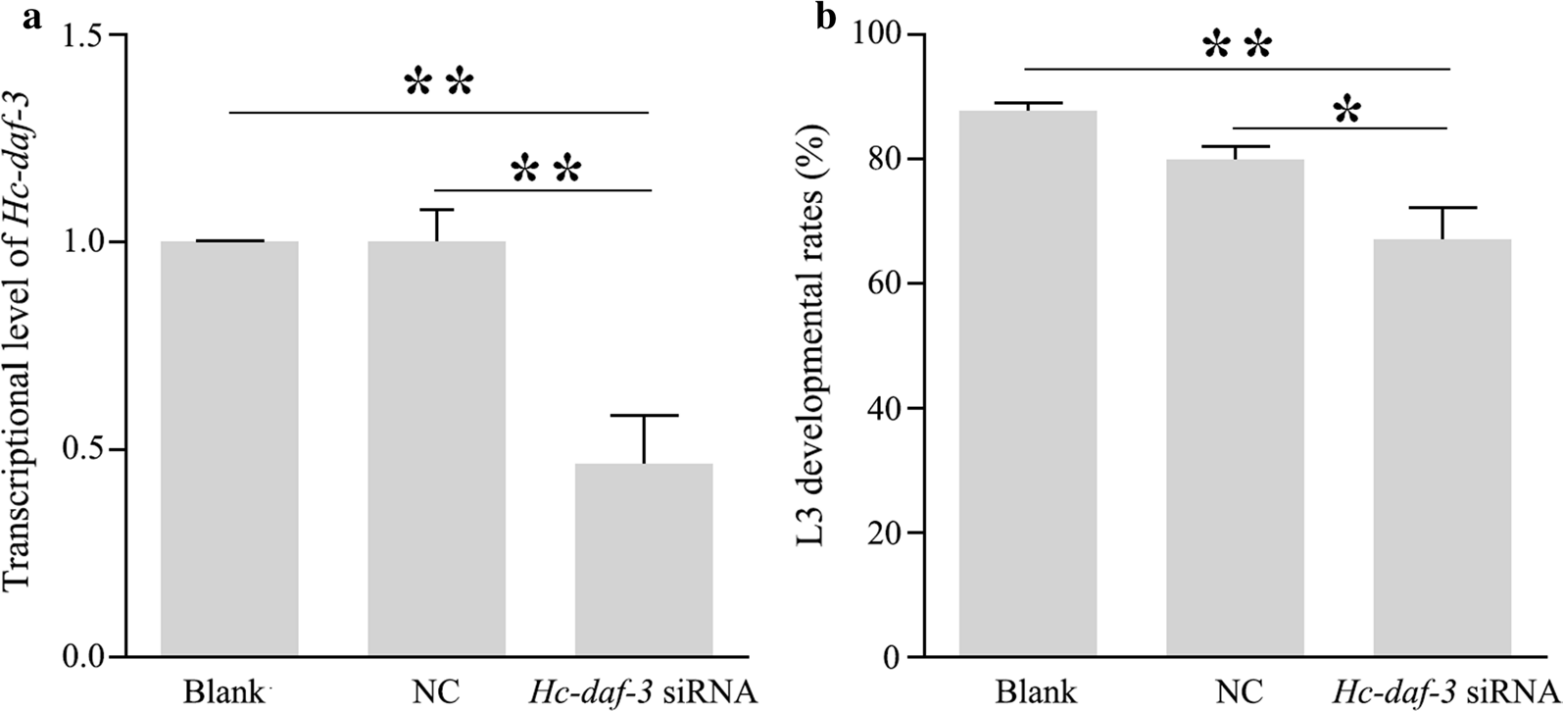
Effects of *Hc-daf-3* siRNA silencing on the development of *Haemonchus contortus*. **a** The transcriptional changes of *Hc-daf-3* in *H. contortus* after RNAi detected by real-time PCR. **b** L3 developmental rates (%) of L4 *in vitro* for further 5 days after RNAi. *P < 0.05, **P < 0.01

During the development of xL3 to L4 of *H. contortus* under *in vitro* conditions, the most obvious changes in morphology occurred in the buccal region. While xL3 showed filled oesophageal tissues, L4 showed well-developed mouthparts (Additional file 3: Figure S2) [26, 27]. After *in vitro* soaking of xL3 of *H. contortus* in *Hc-daf-3* siRNA, transcriptional levels of *Hc-daf-3* in siRNA-treated worms were evaluated using real-time PCR to assess whether *Hc-daf-3* was successfully silenced. The results revealed a significant reduction (53%) in *Hc-daf-3* transcript levels in *Hc-daf-3* siRNA treated xL3 compared with no-siRNA template and irrelevant siRNA controls (*F*_(2, 6)_ = 14.92, *P* = 0.0077 and *P* = 0.0077, respectively) and the transcription of *Hc-daf-3* was similar between the two control groups (Fig. 6a). In addition, fewer xL3s developed to L4s in the *Hc-daf-3* siRNA-treated group compared with the two control groups (*F*_(2, 15)_ = 10.37, *P* = 0.0011 and *P* = 0.0332, respectively), and there was no difference in L4 development between the two control groups (Fig. 6b).

## Discussion

In the present study, a gene (*Hc-daf-3*) encoding a co-Smad protein (*Hc*-DAF-3) in the DAF-7 signalling pathway of *H. contortus* was identified and structurally and functionally characterised. Unlike the upstream molecules in the DAF-7 signalling pathway in nematodes, such as DAF-7 (TGF-β ligand), DAF-1 (TGF-β type I receptor) and DAF-4 (TGF-β type II receptor), which are broadly conserved across nematode phyla including nematodes belonging to Clades I to V, DAF-3 was only found in the nematodes belonging to Clades IV and V [30]. However, although a DAF-3 homologue was identified in *Strongyloides stercoralis* (Clade IV), it could not be differentiated from *Ss*-DAF-8 based on protein alignment and phylogenetic analysis; thus, DAF-3 and DAF-8 homologues of *S. stercoralis* were named *Ss*-SMAD-5, *Ss*-SMAD-7 and *Ss*-SMAD-8 [31]. This may indicate that DAF-3 of Clade V nematodes has undergone deeper evolutionary changes and developed more specific functions than DAF-3 of Clade IV nematodes.

In *H. contortus*, *Hc*-DAF-3 was identified as a typical member of the co-Smad family. Its MH1 domain has relatively high identities to homologues from a range of species, while the MH2 domain is more divergent. As features of Smads, MH1 domain is involved in DNA-binding, whereas MH2 domain plays a role in protein-protein interacting [3]. It is reported that NLS located in the MH1 domain and NES located in the N-terminus of the linker region enable *Hs*-Smad4 shuttling; therefore, the high conservation of NLS and NES between *Hc*-DAF-3 and *Hs*-SMAD4 suggests that *Hc*-DAF-3 may also be engaged in an active nucleocytoplasmic shuttling [32]. For the DAF-7 signalling pathway in *C. elegans*, the *Ce*-DAF-3 MH1 domain can suppress *daf-7* and *daf-8* transcription by binding to their regulatory regions [7]. However, whether *Hc*-DAF-3 can suppress *Hc-daf-7* and *Hc*-*daf-8* transcription needs further exploration. For another important functional domain, SAD (transcriptional activation domain), previous reports suggested that SAD provided transcriptional capability to *Hs*-SMAD4 by presenting surfaces for interaction with transcription partners in *H. sapiens* [33]. In *Hc*-DAF-3, the conservation of SAD is shown with a proline-rich pattern retained in the sequence rather than strict sequence conservation. The high sequence variation in SAD may imply that structural differences evolved to lodge multiple interacting partners required in transcriptional activation. In addition, the phylogenetic trees in the present study indicated the close relationship between co-Smads of *H. contortus* and *A. ceylanicum*, which suggests the functional conservation of DAF-3s of these two species. Nevertheless, the distant relationship between DAF-3s of free-living nematodes and those of parasitic nematodes suggests the functional divergence of DAF-3s of these nematodes with distinct biological divergence.

*Hc-daf-3* exhibits quite different transcriptional profiles compared with those of *Ce-daf-3*. While *Ce-daf-3* was upregulated in L1, its transcription was low in the dauer stage, including dauer entry and dauer exit [34]. The different transcriptional profiles between *Hc-daf-3* and *Ce-daf-3* may indicate their distinct functions. Likewise, the transcript level of *Ce-daf-7* was also upregulated in L1. However, the transcriptional levels of *daf-7* homologues were high in L3s of parasitic nematodes, including *Ancylostoma caninum* [14, 15], *N. brasiliensis* and *H. contortus* [16], *Strongyloides ratti*, *S. stercoralis* and *Parastrongyloides trichosuri* [17, 18], with the exception of *Heligmosomoides polygyrus* and *Trichotrongylus circumcincta*, for which transcription is maximal in adult stages [16]. This may suggest that the parasitic lifestyle has resulted in the convergence of *daf-7* and *daf-3* transcription, independent of phylogeny. The different transcriptional patterns of *Hc-daf-3* and *Ce-daf-3* might suggest that in the evolution of nematode parasitism, compared with their free-living ancestors, parasitic species make use of signalling pathways differently. In addition, the apparent changes in the control of *daf-3* transcription in *H. contortus*, which is quite different from that of *C. elegans*, fit the tenet of EvoDevo that evolution occurs through alternations in controlling developmental genes [35, 36].

Consistent with the high transcription of *Hc-daf-3* in adult females of *H. contortus*, *Hc*-DAF-3 was strongly expressed in gonad organs of the adult worms, suggesting that *Hc-*DAF-3 participates in egg-lying regulation in adult females and reproductive process in adult males. This result is concordant with that of *Ce*-DAF-3, as it functions during embryonic development; early expression of *Ce*-DAF-3 was observed in embryos [37]. Subsequent research also suggested that mutations in *Ce-daf-3* could suppress phenotypes of the egg-laying defect, caused by DAF-7 pathway mutants, such as *daf-7* and *daf-14* mutants [10], further verified *Ce-daf-3*’s function in egg-laying. In addition to the gonad organs, *Hc*-DAF-3 was also strongly expressed in the cuticle of adult worms. In *H. contortus*, the cuticle is mainly proteinous, enabling the worm resistant to harmful substances [38]. Thus, *Hc-daf-3* may also be associated with this process.

In *C. elegans*, *daf-3* plays complex roles in dauer regulation depending on the different environmental and genetic context; for example, *daf-3* mutants are Daf-d at 25 °C but become Daf-c at 27 °C [8], and it is possible that this response protects worms from temperatures at which they are unable to grow. This may indicate *daf-3* functions to facilitate organism flexibility. Thus, it is reasonable to speculate that *Hc-daf-3* may also function differently in regulating L3 development in *H. contortus* upon responses to different environmental signals. To assess the functional importance of *Hc-daf-3* in the L3 development of *H. contortus*, RNAi was performed by soaking xL3 in siRNA under *in vitro* conditions, which specifically decreased the transcription of *Hc-daf-3*. In addition, silencing *Hc-daf-3* retarded the development of xL3 to L4, indicating the roles of *Hc-daf-3* in development related to the transition from the free-living stage (L3) to the parasitic stage (L4). In *C. elegans*, generally, the expression of DAF-7 can inhibit DAF-3 through R-Smads and eventually prevents the dauer formation or promotes dauer exit [37]. Compared with *Ce-daf-3* which inhibits dauer recovery at 25 °C, *Hc-daf-3*’s functions may reverse in L3 exit regulation so that it promotes the transition from xL3 to parasitic stages.

## Conclusions

In this study, we identified one gene encoding a co-Smad (*Hc*-DAF-3) in *H. contortus*, which contains MH1 and MH2 domains, similar to sequences of related co-Smads in other organisms. The transcription of *Hc-daf-3* was detectable in all developmental stages, with higher levels in L3 and adult females. Native *Hc*-DAF-3 was expressed in the gonad and cuticle of adult worms. Silencing *Hc-daf-3* retarded the development of *H. contortus* from xL3 to L4. Collectively, these results provide evidence that *Hc-daf-3* functions in development related to the transition to parasitism in *H. contortus*, and also widen insights into the TGF-β signalling pathway in parasitic nematodes.

## Supplementary information

**Additional file 1: Table S1.** Primers used for PCR-amplification of target gene and for real-time PCR analysis. **Table S2.** Sequences of SMAD4 homologues from all species used for alignment and phylogenetic analysis. **Table S3.** Sequences of *Hc-daf-3*-specific siRNA and control siRNA used for RNA interference. **Table S4.** Sequence identities of *Hc*-DAF-3 and its MH1 domain and MH2 domain relative to homologues from selected metazoan species.

**Additional file 2: Figure S1.** Western blot analysis to detect the native *Hc*-DAF-3 protein from *Haemonchus contortus*. Protein extracts were analysed by SDS-PAGE and transferred onto a PVDF membrane. Western bolt was probed with rabbit antiserum raised against synthetic peptides of *Hc*-DAF-3. **a** Pre-bleed (before immunisation) rabbit serum. **b** Antiserum of *Hc*-DAF-3.

**Additional file 3: Figure S2.** The buccal morphology of xL3 (**a**) and L4 larvae (**b**). Arrows mark the buccal region.

## Data Availability

Data supporting the conclusions of this article are included within the article and its additional file.

## Abbreviations

CDS: coding sequence
DEPC: diethyl pyrocarbonate
L3s: third-stage larvae
ML: maximum likelihood
MP: maximum parsimony
NJ: neighbour-joining
RNAi: RNA interference
siRNA: small interfering RNA
TBS: tris-buffered saline
