# Supplementary material for: A DAF-3 co-Smad molecule functions in Haemonchus contortus development
Source: Parasit Vectors. 2019 Dec 27;12:609. doi: 10.1186/s13071-019-3855-3 (PMC6935219; doi:10.1186/s13071-019-3855-3)
Supplement: Supplementary file 1 — Additional file 1: Table S1. Primers used for PCR-amplification of target gene and for real-time PCR analysis. Table S2. Sequences of SMAD4 homologues from all species used for alignment and phylogenetic analysis. Table S3. Sequences of Hc-daf-3-specific siRNA and control siRNA used for RNA interference. Table S4. Sequence identities of Hc-DAF-3 and its MH1 domain and MH2 domain relative to homologues from selected metazoan species. [file 13071_2019_3855_MOESM1_ESM.docx]

**Additional file 1: Table S1.** Oligonucleotide primers (5’-3’) used in the present study.

| **Primer** | **Sequence (5’-3’)** |
| --- | --- |
| *Hc-daf-3* gene isolating | |
| Hc-daf-3-cF | ATGCGAAGCTCATCCGCTGCTCCCT |
| Hc-daf-3-cR | TCATATTTCATGCATCAGTTCGT |
| Transcription level analysis | |
| Hc-daf-3-qF | TGCGTAGCGCGGATATCATT |
| Hc-daf-3-qR | CATGCATCAGTTCGTCCAAGT |
| Hc-tubulin-qF | TGTTCCATCACCCAAGGTATCC |
| Hc-tubulin-qR | TGACAGACACAAGGTGGTTGAGAT |
| RNA interference in *H. contortus* | |
| Hc-18S-qF | AATGGTTAAGAGGGACAATTCG |
| Hc-18S-qR | CTTGGCAAATGCTTTCGC |

**Additional file 1: Table S2.** Sequences of SMAD4 used for alignment and phylogenetic analysis.

| **Species** | **GenBank**  **accession number** | | **Reference** |
| --- | --- | --- | --- |
| *Ancylostoma ceylanicum* | EYB99633.1 | [1] | |
| *Ascaris suum*^3^ | ERG79533.1 | [2] | |
| *Brugia malayi* | CRZ23865.1 | [3] | |
| *Brugia malayi* | CRZ23866.1 |  |  |
| *Caenorhabditis brenneri* | EGT54842.1 | [4] | |
| *Caenorhabditis briggsae* | XP_002643241.1 | [5] | |
| *Caenorhabditis elegans* | NP_508161.3 | [6] | |
| *Caenorhabditis elegans* | NP_001024604.1 |  |  |
| *Caenorhabditis elegans* | NP_001024605.1 |  |  |
| *Caenorhabditis elegans* | NP_001040864.1 |  |  |
| *Caenorhabditis elegans*^2^ | NP_492321.1 |  |  |
| *Caenorhabditis remanei* | XP_003107018.1 | [7] | |
| *Drosophila melanogaster* | NP_733438.1 | [8] | |
| *Haemonchus contortus* | CDJ96373.1 | [9] | |
| *Homo sapiens*^1^ | AAA91041.1 | [10] | |
| *Loa loa* | EFO19570.2 | [11] | |
| *Mus musculus*^1^ | AAM74472.1 | [12] | |
| *Schistosoma mansoni* | AAQ84177.1 | [13] | |
| *Toxocara canis* | KHN83995.1 | [14] | |

^1^ Sequence was used for alignment analysis.

^2^ Sequence was used as an outgroup for phylogenetic analysis.

^3^ The genomic sequence of this record was removed because it has been superseded by a new assembly of the genome, but the GenBank accession of the new sequence is not available now.

**Additional file 1: Table S3.** Sequences of *Hc-daf-3*-specific siRNA and control siRNA

| **Name** | **Sequence** | **Targeted regions** |
| --- | --- | --- |
| S1 siRNA | Sence: 5’-CCGACAAAUGUGUAACUAUTT-3’ | 374-392 bp |
|  | anti-sense: 5’-AUAGUUACACAUUUGUCGGTT-3’ |  |
| S2 siRNA | Sence: 5’- CCAGCAACAUGCUCAACAATT-3’ | 1062-1080 bp |
|  | anti-sense: 5’-UUGUUGAGCAUGUUGCUGGTT-3’ |  |
| S3 siRNA | Sence: 5’-CCAAAUGGCCAAGGAGUAUTT-3’ | 1839-1857 bp |
|  | anti-sence: 5’-AUACUCCUUGGCCAUUUGGTT-3’ |  |
| Control siRNA | Sence: 5’-UUCUCCGAACGUGUCACGUTT-3’ | Not applicable |
|  | anti-sence: 5’-ACGUGACACGUUCGGAGAATT-3’ |  |

**Additional file 1: Table S4.** Sequence identities of *Hc*-DAF-3 and its MH1 domain and MH2 domain to homologues from selected metazoan species.

| **Homologues** | ***Hc*-DAF-3-MH1** | ***Hc*-DAF-3-MH2** | ***Hc*-DAF-3** |
| --- | --- | --- | --- |
|  | **Identity (%)** | **Identity (%)** | **Identity (%)** |
| *A. ceylanicum*-hypothetical protein | 97.5 | 97.5 | 76.5 |
| *T. canis*-SMAD4 | 77.1 | 41.4 | 45.0 |
| *A. suum*-SMAD4 | 76.2 | 42.4 | 43.3 |
| *B. malayi*-DAF-3 | 75.4 | 45.5 | 42.6 |
| *H. sapniens*-SMAD4 | 70.3 | 43.5 | 39.7 |
| *M. musculus*-SMAD4 | 66.7 | 43.5 | 39.5 |
| *D. melanogaster*-SMAD4 | 67.5 | 43.3 | 35.8 |
| *H. contortus*-SMA-4 | 58.7 | 35.9 | 35.9 |
| *C. elegans*-DAF-3 | 55.6 | 44.3 | 34.1 |
| *C. elegans*-SMA-4 | 58.7 | 35.0 | 34.1 |
| *S. mansoni*-SMAD4 | 55.0 | 40.6 | 31.4 |

**References**

1. Schwarz EM, Hu Y, Antoshechkin I, Miller MM, Sternberg PW, Aroian RV. The genome and transcriptome of the zoonotic hookworm *Ancylostoma ceylanicum* identify infection-specific gene families. Nat Genet. 2015;47:416–22.

2. Wang J, Czech B, Crunk A, Wallace A, Mitreva M, Hannon GJ, et al. Deep small RNA sequencing from the nematode *Ascaris* reveals conservation, functional diversification, and novel developmental profiles. Genome Res. 2011;21:1462–77.

3. Ghedin E, Wang S, Spiro D, Caler E, Zhao Q, Crabtree J, et al. Draft genome of the filarial nematode parasite *Brugia malayi*. Science. 2007;317:1756–60.

4. Mulder NJ, Apweiler R, Attwood TK, Bairoch A, Barrell D, Bateman A, et al. The InterPro Database, 2003 brings increased coverage and new features. Nucleic Acids Res. 2003;31:315–8.

5. Stein LD, Bao Z, Blasiar D, Blumenthal T, Brent MR, Chen N, et al. The genome sequence of *Caenorhabditis briggsae*: a platform for comparative genomics. PLoS Biol. 2003;1:E45.

6. Consortium CeS. Genome sequence of the nematode *C. elegans*: a platform for investigating biology. Science. 1998;282:2012–8.

7. Bieri T, Blasiar D, Ozersky P, Antoshechkin I, Bastiani C, Canaran P, et al. WormBase: new content and better access. Nucleic Acids Res. 2007;35(Database issue):D506–10.

8. Matthews BB, Dos Santos G, Crosby MA, Emmert DB, St Pierre SE, Gramates LS, et al. Gene model annotations for *Drosophila melanogaster*: impact of high-throughput data. G3 (Bethesda). 2015;5:1721–36.

9. Laing R, Kikuchi T, Martinelli A, Tsai IJ, Beech RN, Redman E, et al. The genome and transcriptome of *Haemonchus contortus*, a key model parasite for drug and vaccine discovery. Genome Biol. 2013;14:R88.

10. Hahn SA, Schutte M, Hoque AT, Moskaluk CA, da Costa LT, Rozenblum E, et al. DPC4, a candidate tumor suppressor gene at human chromosome 18q21.1. Science. 1996;271:350–3.

11. Desjardins CA, Cerqueira GC, Goldberg JM, Dunning Hotopp JC, Haas BJ, Zucker J, et al. Genomics of *Loa loa*, a *Wolbachia*-free filarial parasite of humans. Nat Genet. 2013;45:495–500.

12. Hohenstein P, Molenaar L, Elsinga J, Morreau H, van der Klift H, Struijk A, et al. Serrated adenomas and mixed polyposis caused by a splice acceptor deletion in the mouse *Smad4* gene. Genes Chromosomes Cancer. 2003;36:273–82.

13. Osman A, Niles EG, LoVerde PT. Expression of functional *Schistosoma mansoni* Smad4: role in Erk-mediated transforming growth factor beta (TGF-beta) down-regulation. J Biol Chem. 2004;279:6474–86.

14. Zhu XQ, Korhonen PK, Cai H, Young ND, Nejsum P, von Samson-Himmelstjerna G, et al. Genetic blueprint of the zoonotic pathogen *Toxocara canis*. Nat Commun. 2015;6:6145.
