# Supplementary figures and images for: A DAF-3 co-Smad molecule functions in Haemonchus contortus development
Source: Parasit Vectors. 2019 Dec 27;12:609. doi: 10.1186/s13071-019-3855-3 (PMC6935219; doi:10.1186/s13071-019-3855-3)

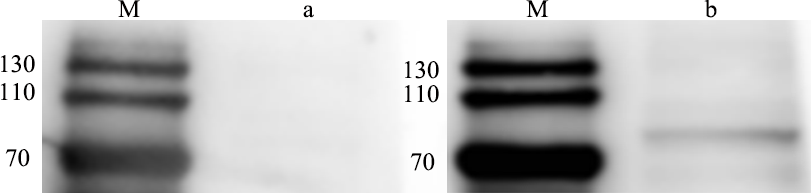

Supplement: Supplementary file 2 — Additional file 2: Figure S1. Western blot analysis to detect the native Hc-DAF-3 protein from Haemonchus contortus. Protein extracts were analysed by SDS-PAGE and transferred onto a PVDF membrane. Western bolt was probed with rabbit antiserum raised against synthetic peptides of Hc-DAF-3. a Pre-bleed (before immunisation) rabbit serum. b Antiserum of Hc-DAF-3. [file 13071_2019_3855_MOESM2_ESM.tif]
